# Distinct fronto-striatal couplings reveal the double-faced nature of response–outcome relations in instruction-based learning

**DOI:** 10.3758/s13415-014-0325-4

**Published:** 2014-11-01

**Authors:** Hannes Ruge, Uta Wolfensteller

**Affiliations:** Department of Psychology, Technische Universität Dresden, Dresden, Germany

**Keywords:** Instruction-based learning, Model-based learning, Goal-directed action, Differential outcomes, Fronto-striatal coupling, Fronto-hippocampal coupling, Ideomotor theory

## Abstract

Higher species commonly learn novel behaviors by evaluating retrospectively whether actions have yielded desirable outcomes. By relying on explicit behavioral instructions, only humans can use an acquisition shortcut that prospectively specifies how to yield intended outcomes under the appropriate stimulus conditions. A recent and largely unexplored hypothesis suggests that striatal areas interact with lateral prefrontal cortex (LPFC) when novel behaviors are learned via explicit instruction, and that regional subspecialization exists for the integration of differential response–outcome contingencies into the current task model. Behaviorally, outcome integration during instruction-based learning has been linked to functionally distinct performance indices. This includes (1) compatibility effects, measured in a postlearning test procedure probing the encoding strength of outcome–response (O–R) associations, and (2) increasing response slowing across learning, putatively indicating active usage of O–R associations for the online control of goal-directed action. In the present fMRI study, we examined correlations between these behavioral indices and the dynamics of fronto-striatal couplings in order to mutually constrain and refine the interpretation of neural and behavioral measures in terms of separable subprocesses during outcome integration. We found that O–R encoding strength correlated with LPFC–putamen coupling, suggesting that the putamen is relevant for the formation of both S–R habits and habit-like O–R associations. By contrast, response slowing as a putative index of active usage of O–R associations correlated with LPFC–caudate coupling. This finding highlights the relevance of the caudate for the online control of goal-directed action also under instruction-based learning conditions, and in turn clarifies the functional relevance of the behavioral slowing effect.

**Electronic supplementary material:**

The online version of this article (doi:10.3758/s13415-014-0325-4) contains supplementary material, which is available to authorized users.

Higher animals, including humans, commonly learn novel behaviors via trial and error, by evaluating retrospectively whether an action performed under certain stimulus conditions yielded desirable outcomes. However, only humans are able to cut short this acquisition process by relying on explicit behavioral instructions that specify prospectively how to yield an intended outcome. This enables humans to directly yield the intended outcomes while circumventing potentially harmful trial-and-error learning (Doll, Jacobs, Sanfey, & Frank, 2009). Although the great importance of this issue was acknowledged early on (Duncan, Emslie, Williams, Johnson, & Freer, 1996; Luria, 1973; Monsell, 1996; Noelle, 1997), the underlying mechanisms have still been little explored (Cole, Laurent, & Stocco, 2012; Wolfensteller & Ruge, 2012). Ultimately, learning by feedback and learning by instruction both enable the expression of goal-directed action by integrating anticipated response outcomes already during action selection. As a prerequisite, organisms need to learn that a certain outcome O is obtained by a certain response R (response–outcome contingency) under the appropriate stimulus conditions (S).

## Fronto-striatal interactions during instruction-based learning

Under feedback-driven trial-and-error learning conditions, it is widely believed that distinct striatal substructures are important for establishing either outcome-directed (i.e., goal-directed) action relying on the anterior caudate or habit-like (i.e., directly stimulus-triggered) action relying on the putamen (Ashby, Turner, & Horvitz, 2010; Dolan & Dayan, 2013; Seger & Spiering, 2011; Yin & Knowlton, 2006). The picture gains complexity when considering that the striatum is not operating in isolation. Neuro-anatomically, it is clear that the striatum is heavily interconnected with the prefrontal cortex (PFC; Alexander, DeLong, & Strick, 1986). Hence, one might expect that both areas engage interactively when learning and behavior is “model-based” rather than “model-free” in the sense that it is driven by explicit knowledge of contingencies stored in lateral PFC (LPFC) working memory (cf. Dolan & Dayan, 2013; Glascher, Daw, Dayan, & O’Doherty, 2010). This view has recently been expressed in a few formal models on instruction-based control of learning and behavior (Doll et al., 2009; Huang, Hazy, Herd, & O’Reilly, 2013; Ramamoorthy & Verguts, 2012). Moreover, recent neuroimaging studies have demonstrated that LPFC and striatal areas become functionally coupled when learning is under the control of explicitly instructed rules (Li, Delgado, & Phelps, 2011; Ruge & Wolfensteller, 2013). Such findings support the notion that model-based learning and model-free feedback-driven learning might share the same striatal machinery (cf. Daw, Gershman, Seymour, Dayan, & Dolan, 2011; Doll, Simon, & Daw, 2012). Furthermore, first evidence also suggests a similar striatal functional differentiation in that learning-related dynamics of LPFC–anterior caudate coupling, but not LPFC–putamen coupling, is sensitive to the manipulation of response–outcome contingency during instructed stimulus–response learning (Ruge & Wolfensteller, 2013). Hence, specifically LPFC–anterior caudate coupling seems to reflect processes linked to the integration of response–outcome contingencies into an explicit model of the current task.

The primary aim of the present study was to further refine the functional interpretation of this basic finding regarding LPFC–caudate coupling by interrelating functional connectivity measures with two distinct performance measures that have been identified in behavioral studies and might reflect distinct subprocesses of outcome integration during stimulus-based response selection.

In particular, it is commonly thought that goal-directed action selection is enabled by integrating outcomes via an S–O → O–R activation chain, both according to ideomotor theory and instrumental learning theory (Balleine & Ostlund, 2007; de Wit & Dickinson, 2009; Urcuioli, 2005). In our previously reported experimental setup (Ruge & Wolfensteller, 2013), it was impossible to decide whether the observed LPFC–caudate coupling effects indicated O–R association encoding by itself or rather the active usage of increasingly learned O–R associations through the S–O → O–R chain. In the present study, we therefore added a test phase after each S–R–O learning phase, in which previous outcomes now served as imperative stimuli that required responses that could be either compatible or incompatible with the previously acquired O–R associations. Thereby, we were able to obtain a relatively pure measure of O–R association strength.

As in our previous design, S–R–O learning was induced via the introduction of differential response outcomes during instructed S–R learning, which means that each distinct S–R link was followed by a distinct outcome (Colwill & Rescorla, 1985; de Wit & Dickinson, 2009; Noonan, Mars, & Rushworth, 2011; Shin, Proctor, & Capaldi, 2010; Trapold, 1970; Urcuioli, 2005). Typically, in trial-and-error learning situations this “differential outcome effect” is evidenced by a more rapid decline of error rates from initial chance-level performance when comparing differential outcome conditions to common or random outcome conditions (Trapold, 1970; Urcuioli, 2005) and when the S–R learning task is sufficiently demanding (Estévez, Fuentes, Mari-Beffa, González, & Alvarez, 2001; Legge & Spetch, 2009). In instruction-based learning, which is characterized by an extremely low error rate from the start, this error rate effect clearly cannot serve as an adequate marker of outcome integration. But S–R learning that is too easy to show any further benefit of differential outcomes on error rates is nevertheless associated with significant response-slowing across learning when compared to a common outcome condition (Ruge, Krebs, & Wolfensteller, 2012). Analogous to the accelerated error rate decline in demanding trial and error learning situations, response slowing during instruction-based S–R–O learning can be interpreted as a putative index of active O–R usage for the online control of goal-directed action selection. This is generally consistent with the notion that differential outcomes are rapidly integrated into an extended model of the current task instruction mediated via the “fast-learning but slow-acting” prefrontal cortex control system (cf. Dolan & Dayan, 2013).^1^ In the present study, we tested the hypothesis that such quickly established prefrontal S–R–O representations enable goal-directed action selection via functional couplings with the anterior caudate nucleus.

Besides O–R-related response slowing across S–R–O learning, which we suggest is indicative of active O–R usage, numerous studies conducted in the realm of ideomotor theory (Greenwald, 1970) have shown that the strength of O–R encoding by itself is expressed in O–R compatibility effects measured in a subsequent outcome-priming test phase (for a review, see Shin et al., 2010). Such O–R compatibility effects are taken to indicate that perceiving a previous outcome activates automatically or unintentionally the action that it was previously produced by and that can be either compatible or incompatible with the current test-phase response requirements (Elsner & Hommel, 2001; Shin et al., 2010). Importantly, this effect can be observed even after very short acquisition periods as in the present study (Ruge et al., 2012; Wolfensteller & Ruge, 2011). The size of the O–R compatibility effect can hence serve as an index of “O–R encoding” analogously to previous fMRI studies based on the “subsequent memory” rationale, in which postlearning behavioral indices are correlated with brain activation recorded during learning (Brewer, Zhao, Desmond, Glover, & Gabrieli, 1998; Summerfield et al., 2006). Hence, on the basis of this additional O–R compatibility index we aimed to determine the specificity of the hypothesized LPFC–caudate coupling for active O–R usage rather than O–R encoding by itself. Moreover, using a similar rationale, previous ideomotor learning studies reported activations in the hippocampus when extensively learnt response outcomes served as primes that should automatically trigger response tendencies consistent with previously established O–R associations (Elsner et al., 2002; Melcher et al., 2013). Different from these studies that investigated highly overlearned O–R associations, we hypothesized that hippocampal O–R encoding under instruction-based learning conditions and especially during an early phase of learning might depend on the functional coupling with the LPFC (Huang et al., 2013).

## Method

### Subjects

Twenty-eight human subjects participated in this study. Four of the subjects were excluded due to high error rates exceeding 25 % on the first unguided implementation trial (see below), suggesting that the instructions were not well memorized, thereby inducing a strong (undesired) trial-and-error learning component. An additional subject was excluded due to early abandoning of the experiment. The mean age of the remaining 23 subjects was 24.2, ranging between 18 and 31 years (12 female, 11 male). The experimental protocol was approved by the Ethics Committee of the Technische Universität Dresden in agreement with the World Medical Association’s Declaration of Helsinki. All subjects gave written informed consent prior to taking part in the experiment and were paid €8 per hour or received course credit.

### Materials and procedure

#### S–R–O learning phase

**Fig. 1:**
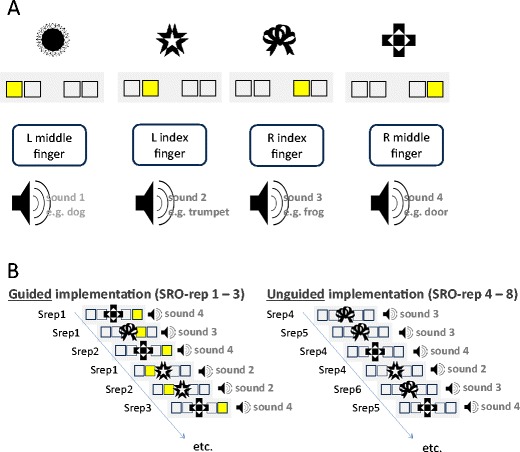
Schematic representation of the S–R–O learning phase. (A) Exemplary mapping between stimuli (visual shapes), instruction cues (colored squares), responses, and sound outcomes. During the experiment, subjects had to learn 11 different mappings, each involving novel visual stimuli and novel sound outcomes. (B) A novel S–R–O mapping was learned through explicit instruction provided by the instruction cues presented in the “guided implementation phase,” comprising the first three repetitions of each of the four S–R–O triples. During the “unguided implementation phase” (S–R–O Repetitions 4–8), instruction cues were no longer presented, and hence the correct response had to be retrieved from memory.

We employed a modified version of our original instruction-based learning paradigm (Ruge & Wolfensteller, 2010). Instructions were delivered via a “guided implementation” procedure in which the instruction was embedded within the first few behavioral implementation trials, which also comprised the presentation of differential outcomes following correct responses (see Fig. 1). The guided implementation phase comprised 12 correct trials, including three repetitions of four distinct S–R–O triples (termed *S–R–O repetition* in the following discussion). A trial started with the presentation of the visual stimulus S for 500 ms. The visual stimuli were four distinct abstract shapes. Following 250 ms after S onset, an additional instruction stimulus (IS) was displayed, which remained on screen until a response was made or until timeout after 1,750 ms. The IS was a yellow square highlighting one of four constantly displayed empty boxes. The manual responses (left middle finger, left index finger, right index finger, and right middle finger) were mapped in a spatially compatible manner to the IS position. The differential outcomes were four distinct naturalistic sounds and were emitted for 500 ms directly following a correct response. This guided implementation phase was followed by an unguided implementation phase in which the IS was omitted, which comprised another 20 correct trials (i.e., five repetitions of the four distinct S–R–O triples). Hence, starting from the fourth S–R–O repetition, the correct response had to be retrieved from memory, since it was not indicated by the IS anymore. Erroneous trials were directly repeated in both learning phases, and the outcome was replaced by the German word for “error.” The experiment comprised 11 such S–R–O learning blocks, each with novel visual stimuli and novel outcome sounds. The intertrial interval (ITI) was randomly selected from a distribution including interval durations of 800 ms (24 trials per block), 2,350 ms (five trials per block), and 4,700 ms (three trials per block). Analyses of learning-related changes in behavior and brain activation were based on SRO repetitions, with levels ranging between SRO-Rep1 and SRO-Rep8.

#### O–R test phase

**Fig. 2:**
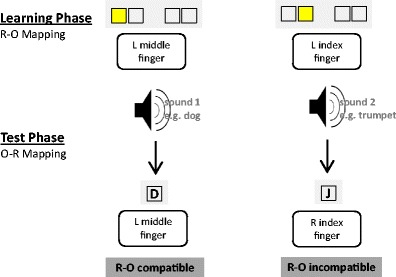
Schematic representation of the O–R test phase. The four sound stimuli that had been produced by certain responses in the S–R–O learning phase now served as imperative stimuli in the test phase. The required response could be the same (compatible) or different (incompatible), with respect to the response that had produced that sound beforehand. The correct response was indicated by the instruction cues (letters D, F, J, and K) during the first three repetitions of each of the four sound–response pairs. Starting from the fourth repetition, the correct response had to be retrieved from memory. Test-phase data were solely used to compute the size of the behavioral O–R compatibility effect, as an index of O–R strength, which was then correlated with the brain activation data collected during the preceding S–R–O learning phase.

Each of the 11 S–R–O learning blocks was followed by a test phase probing the strength of the just-encoded O–R associations, similar to the typical testing procedures used in the context of ideomotor theory (Shin et al., 2010). The previous outcome sounds were now serving as stimuli that again required one of the four responses (see Fig. 2). The responses keys were the same as during the preceding S–R–O learning phase. Two outcome sounds were mapped to the response that had produced that sound in the preceding phase (*compatible* trials), whereas the two remaining outcome sounds were mapped to responses that had produced another sound before (*incompatible* trials). Hence, according to ideomotor theory, previously learned O–R associations should prime the correct response in compatible trials, and the incorrect response in incompatible trials. As in the preceding S–R–O learning phase, the novel sound–response mappings were learned via the two-step instruction procedure, comprising 12 guided and 20 unguided implementation trials. The instruction stimuli (ISs) were now the letters D, F, J, and K, presented centrally on the screen and mapped onto the left middle finger, left index finger, right index finger, and right middle finger, respectively. Different IS types were used in the learning and test phases, to deconfound O–R and IS–R compatibility. The nonspatial IS–response mapping was practiced outside the scanner until performance accuracy was greater than 90 % in two consecutive blocks of 20 trials. A trial started with a fixation cross displayed for 500 ms, followed by the sound, lasting 500 ms. In the guided phase, the IS was presented 150 ms after sound onset and lasted until the response or until timeout after 1,500 ms. Accuracy feedback was displayed for 650 ms, indicating correct, wrong, or too-slow responses. The ITI distribution was the same as in the S–R–O learning phase. Note that the test-phase data were exclusively used to extract the behavioral O–R compatibility index, based on the unguided implementation trials. This index then served as a covariate in the fMRI analysis of the S–R–O learning-phase brain activation data. Before entering the scanner, subjects completed one S–R–O learning block and one O–R test block to familiarize them with the task procedure.

### FMRI recording

Whole-brain images were acquired on a Siemens 3-T whole-body Trio System (Erlangen, Germany) with a 16-channel circularly polarized head coil. Headphones (MR-Confon) dampened the scanner noise and were used for sound presentation. Both structural and functional images were acquired for each subject. High-resolution structural images (1.0 × 1.0 × 1.0 mm) were acquired using an MP-RAGE T1-weighted sequence (TR = 1,900 ms, TE = 2.26 ms, TI = 900 ms, flip = 9°). Functional images were acquired using a gradient echo-planar sequence (TR =2,000 ms, TE =30 ms, flip =80°). Each volume contained 32 4.0-mm-thick slices (in-plane resolution 4.0 ×4.0 mm) plus 0.8-mm gap. Slices were oriented parallel to the AC–PC plane. The experiment was controlled by E-Prime 2.0 software running on a Windows XP PC. Visual stimuli were displayed by an LCD projector on a back-projection screen mounted behind the magnet. Subjects viewed the screen through mirror glasses. A fiber-optic, light-sensitive keypress device was used to record subjects’ behavioral responses.

### fMRI preprocessing

The fMRI data set was analyzed with SPM8 running under MATLAB 8.0.0.783. Preprocessing included slice-time correction, rigid body movement correction (three translation, three rotation parameters), and normalization of the functional images by directly registering the mean functional image to the standard Montreal Neurological Institute (MNI) echo-planar-imaging template image provided by SPM8, with a resulting interpolated spatial resolution of 3 × 3 × 3 mm. Finally, images were spatially smoothed (Gaussian kernel, full width at half maximum = 8 mm). During model estimation, a temporal high-pass filter with a cutoff frequency of 1/256 Hz was applied.

### fMRI analysis

#### First-level analysis

For the analysis of the functional couplings between LPFC and any other voxel in the brain, we used the psychophysiological interaction (PPI) framework implemented in SPM8 (Friston et al., 1997; Gitelman, Penny, Ashburner, & Friston, 2003) in its generalized form proposed recently (McLaren, Ries, Xu, & Johnson, 2012). Essentially, the PPI method predicts the blood-oxygenation-level-dependent (BOLD) signal time course in “target” voxels anywhere in the brain volume, on the basis of the BOLD signal time course in a prespecified “seed” voxel. Importantly, functional coupling is not simply defined by a significant regression between two BOLD signal time courses, but it is defined in terms of changes in regression slopes as a function of varying psychological states associated with two experimental conditions A versus B. The generalized PPI used here accommodates situations in which the two contrasted conditions are occurring in the context of additional experimental conditions intermixed with the conditions of interest. For each experimental condition two regressors were included in a generalized linear model (GLM). This includes a standard event-related regressor obtained by convolving a canonical BOLD response model with a stick function representing the repeated occurrences of that condition. This “task regressor” picks up the average event-related BOLD activation level associated with that condition. The second regressor is obtained by multiplying the task regressor with the seed voxel activation time course (by first deconvolving the seed voxel activation time course, then multiplying with the task-related stick function, and then reconvolving with the canonical BOLD response, all as described by Gitelman et al., 2003). In linear combination with the task regressor, this “PPI regressor” picks up any trial-to-trial deviation from the mean task-related activation that is common between seed voxel and target voxel. Hence, the PPI regressor measures task-related synchrony between the seed-voxel and target-voxel activation time courses, which defines the term “functional coupling” used in the present article. Finally, the GLM comprises the seed voxel time course as an additional regressor to bind unspecific sources of covariance between seed voxel and target voxel. In the present study, we analyzed PPI regressor estimates for early learning trials (SRO-Rep2 and SRO-Rep3) and late learning trials (SRO-Rep7 and SRO-Rep8). Note that we did not include SRO-Rep1 within the category of early learning trials as this was considered to be a very special condition comprising a strong perceptual novelty component due to the fact that visual stimuli and sounds were perceived for the very first time. Hence, we considered SRO-Rep2 and SRO-Rep3 to be more “neutral” instances of early learning trials.

#### Second-level analysis

The primary goal of the present study was to determine possible correlations between the two putative behavioral measures of O–R encoding strength and O–R usage, on the one hand, and the functional coupling between lateral PFC and other brain structures, on the other hand. We therefore included both behavioral indices as covariates in the group-level analysis of PPI effects. By simultaneously including both covariates, we made sure that a correlation between PPI effect and one covariate was uniquely driven by a covariance component that was orthogonal to the other covariate. In other words, we determined partial correlations between the PPI effect and one covariate, with the potential influence of the other covariate being regressed out. Note that this procedure does not exclude the possibility that both covariates might be significantly correlated with the same PPI effect in the same target voxel. Yet, these correlations would then be due to different covariance sources. To make sure that we would not miss any significant correlation due to covariance components common to both covariates, we additionally computed group-level analyses that comprised only one of the two covariates, respectively (to foreshadow the results: We did not observe such additional effects). Furthermore, we ran complementary control analyses that included error rate changes across learning as an additional covariate (see below, Step 3 of the analysis).

The covariance analysis proceeded in three steps. We first identified for each covariate significant correlations with the PPI effect defined by the difference in functional couplings in late learning trials relative to early learning trials. Significant activations were required to exceed a family-wise-error (FWE) threshold *p* < .05, adjusted for the volume of each selected region of interest (ROI) either on the voxel level or on the cluster level at a cluster-forming threshold of *p* < .001 (see below for our ROI selection). To search for significant effects outside of the preselected ROIs, we also computed FWE-corrected whole-brain statistics.

In a second step, we performed post-hoc tests to determine whether a significant activation identified in Step 1 was linked to correlations between the behavioral covariate and functional couplings in early learning trials, mid learning trials (SRO-Rep5 and SRO-Rep6), and late learning trials.

In a third step, we ran two complementary control analyses including the subject-wise error rate difference between the late and early learning trials as an additional covariate, either in combination with the covariate for O–R encoding strength or in combination with the covariate for O–R usage. These final analyses were performed in order to control for potential confounding effects between the indices for O–R encoding strength and O–R usage, on the one hand, and error rate differences across S–R–O learning, on the other hand. Even though the mean error rates early and late in learning were low (1.8 % and 4.8 %, respectively, and given an error rate of 75 % for chance performance), this control analysis nevertheless seemed warranted, since error rates varied considerably across subjects (ranging between 0 % and 9 % early and between 0 % and 14 % late).

One possible confounding might be that relative response slowing was directly related to difficulties in instructed S–R translation rather than to the extent of increasing O–R usage. Accordingly, higher error rates late in learning could potentially be associated with slower responding due to higher uncertainty about which response alternative to select given a particular stimulus (since the correct response might not yet be recalled with certainty).

Another possible confounding might be that functional couplings associated with O–R encoding strength might primarily engage in error-driven S–R learning, which might as a side effect also drive O–R learning (possibly even elsewhere in the brain).

#### Regions of interest

Analogously to Ruge and Wolfensteller (2013), and in line with other studies on instruction-based learning (Dumontheil, Thompson, & Duncan, 2011; Hartstra, Kuhn, Verguts, & Brass, 2011), the left posterior LPFC was selected as a seed region (MNI coordinates: –42 8 32). Likewise analogously to Ruge and Wolfensteller (2013), this voxel was selected on the basis of a standard univariate analysis that identified the strongest learning-related activation changes within posterior LPFC via an *F* test computing the main effect across all eight S–R–O repetition levels, with *p*(*F*) < .05, FWE-corrected on the whole-brain level.

We looked for significant PPI effects in three ROIs, including most importantly the left and right basal ganglia as the regions of primary theoretical interest. Additionally, we included the left and right hippocampus, which has been reported in previous studies on instruction-based control (Li et al., 2011; Ruge & Wolfensteller, 2013) and has been suggested to play a critical role in such processes in a formal neurocomputational model (Huang et al., 2013). As a third ROI, the orbitofrontal cortex (OFC) was included, defined by Brodmann areas BA11 and BA47. We included this region because previous studies had suggested its importance in goal-directed action control (e.g., Noonan, Kolling, Walton, & Rushworth, 2012; Ruge & Wolfensteller, 2013; Valentin, Dickinson, & O’Doherty, 2007). Anatomical information was taken from the automatic anatomic labeling atlas (AAL) for the basal ganglia and hippocampus (Tzourio-Mazoyer et al., 2002) and from the Brodmann parcellation used in the MRICRON software for the OFC ROI (Rorden, Karnath, & Bonilha, 2007).

## Results

### Behavior (S–R–O learning phase)

**Fig. 3:**
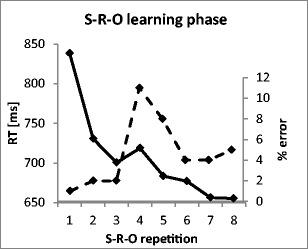
Behavioral data from the S–R–O learning phase. The solid line represents mean response times (RT), and the dashed line represents mean percent error rates. Correct responses were guided by an instruction cue in S–R–O Repetitions 1–3. The instruction cue was omitted in S–R–O Repetitions 4–8.

We computed two separate one-way repeated measures analyses of variance (ANOVAs) with the factor S–R–O Repetition (SRO-Rep1 through SRO-Rep8) for mean response times (RTs) and mean error rates using SPSS 21. The analyses revealed highly significant S–R–O repetition effects for both RTs [*F*(3, 63) = 67.3, *p*(*F*) < .001, Greenhouse–Geisser corrected] and errors [*F*(3, 70) = 15.1, *p*(*F*) < .001, Greenhouse–Geisser corrected]. As expected, we observed a sharp drop in RTs from SRO-Rep1 to SRO-Rep2, likely reflecting a strong “orienting response” upon the introduction of novel stimuli at SRO-Rep1 (see Fig. 3). This was followed by a slight RT increase at SRO-Rep4, indicating the transition into the unguided phase, in which responses had to be selected on the basis of memorized S–R association from the preceding guided learning phase. Then, RTs gradually declined until SRO-Rep8. The learning curve for error rates is mainly characterized by a sharp increase in errors at SRO-Rep4, again indicating the transition into memory-based response selection. Notably, the group mean was 11 % errors at SRO-Rep4, which quickly dropped down to 5 % (see Fig. 3). This indicates that the S–R instruction was well learned, given an expected error rate of 75 % for chance-level performance, due to the four independent and equally probable response choices. The subject-wise behavioral index of active O–R usage entered into the fMRI covariance analysis was defined as the individual RT difference between late learning trials (the mean of SRO-Rep7 and SRO-Rep8) and early learning trials (the mean of SRO-Rep2 and SRO-Rep3). Note that on the mean group level, a significant mean response speed-up was indicated by a negative mean difference between SRO-Rep7/8 and SRO-Rep2/3. Hence, as in previous experiments (Ruge et al., 2012), the O–R-related relative slowing effect was supposed to be embedded in a general speeding effect. This overall speeding effect likely reflects increasingly fluent S–R translation associated with decreasing error rates, which indicates decreasing response uncertainty as fewer response alternatives have to be considered (see also the third step of the second-level fMRI analysis, in the Method section).^2^ To determine the extent to which intersubject variability in response slowing might in fact be due to variability in the S–R-related speed up rather than in O–R-related slowing, we computed the correlation between the RT differences (late – early) and the error rate differences (late – early). This correlation was, however, close to zero, with *r* = .033 (*p* < .88, two-sided), suggesting that variability in response slowing cannot be reduced to the degree of uncertainty in S–R translation, expressed in error rates. In fact, this nonsignificant result might be due to the relatively tiny error rates and error rate changes from early to late in learning, which might hence affect RTs only very marginally.

### Behavior (test phase)

We computed two one-sided paired *t* tests to determine the O–R compatibility effect (incompatible > compatible) for RTs and mean error rates collected during the unguided test phase. The analyses revealed highly significant O–R compatibility effects for RT [*t*(22) = 3.1, *p*(*t*) < .005] and a just-significant effect also for errors [*t*(22) = 1.7, *p*(*t*) < .05]. The subject-wise behavioral index of O–R encoding strength that entered the fMRI covariance analysis was defined as the individual O–R compatibility effect in RT. The correlation between the O–R compatibility effect in the test phase and SRO-related response slowing during the preceding S–R–O learning phase was not significant (*r* = –.28, *p*(*r*) < .20, two-sided). Notably, this is a deviation from previous results, in which we did find significant positive correlations between slowing and the subsequent O–R compatibility effect (Ruge et al., 2012). Currently, it remains elusive which of the various design differences between the present study and the previous study might be responsible for the diverging results.

We also computed the correlation between O–R compatibility effect and the error rate difference during learning, to assess the potential confound between feedback-driven S–R learning and O–R encoding (see also the third step of the second-level fMRI analysis, in the Method section). This correlation was positive but nonsignificant, with *r* = .32 (*p* < .14, two-sided), suggesting that the O–R compatibility effect was not substantially related to uncertainty in S–R translation, expressed in the error rates. Note that during the fMRI analysis we nevertheless performed a control analysis including error rate as an additional covariate, to partial out any common covariance between O–R compatibility, error rate difference, and brain activation.

### fMRI

We first assessed general changes in functional couplings with the LPFC seed from early to late S–R–O learning trials. This analysis replicated previous findings by Ruge and Wolfensteller (2013). Specifically, we found increasing functional couplings between LPFC and anterior caudate, as well between LPFC and OFC (Table 1).

**Table 1:** Functional coupling with LPFC during S–R–O learning (late > early)

Region of interest	Subregion	MNI coordinates			Coupling at late – early			
		*x*	*y*	*z*	*t*	*p* Voxel (FWE-Corr.)	Cluster Size	*p* Cluster (FWE-Corr.)
Left basal ganglia	Ant. caudate	–12	20	–8	4.47	.028	8	.059
Left orbitofrontal	Lateral OFC (BA47)	–42	47	–8	4.45	.060	32	.032
	Lateral OFC (BA47)	–36	41	–8	4.10	.112	Same cluster as above	
Right orbitofrontal	Medial/central OFC (BA11)	18	56	–5	4.69	.042	3	.180

**Fig. 4:**
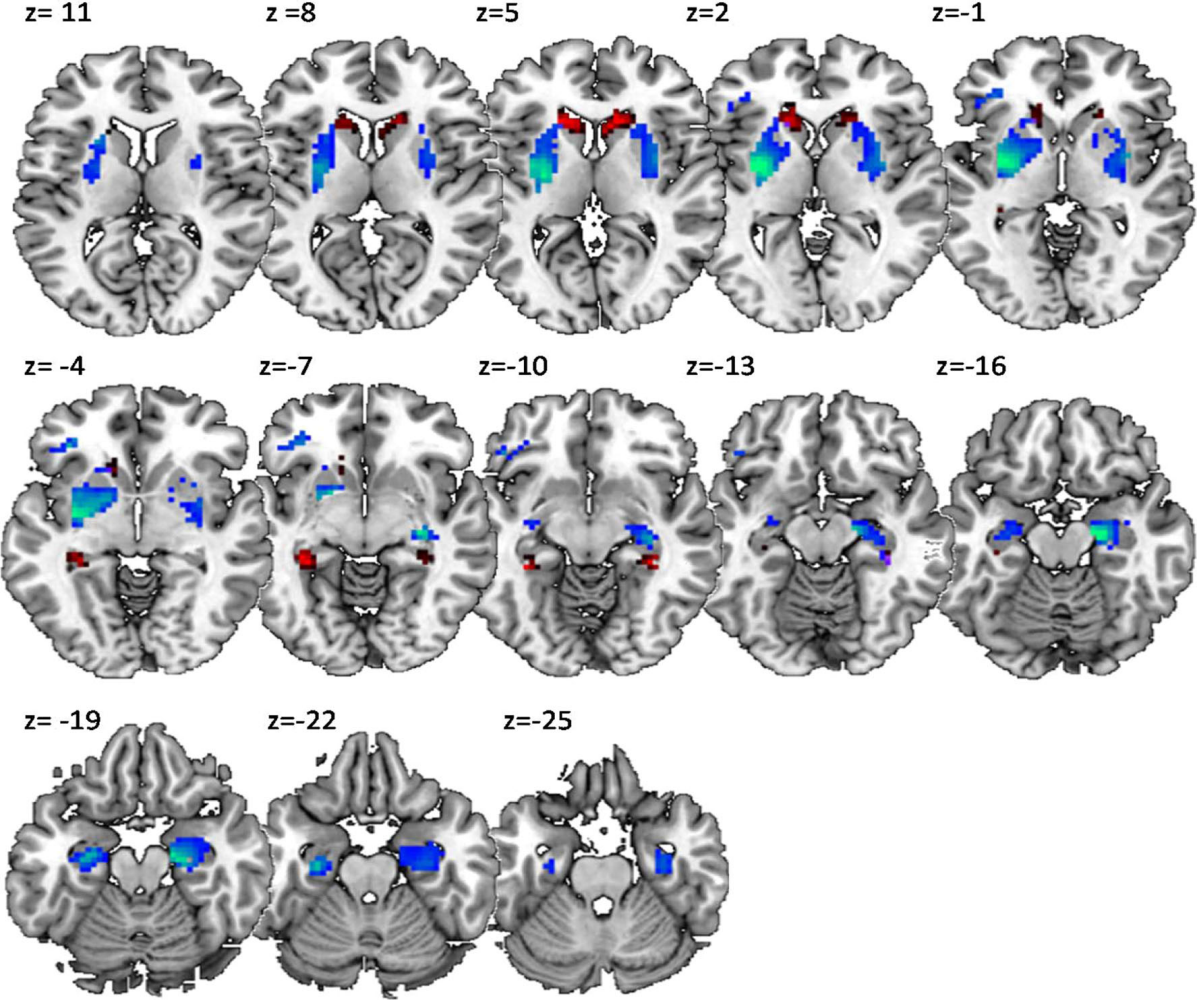
Visualization of the results obtained by the left lateral-prefrontal-cortex-seeded (MNI –42 8 28) psychophysiological interaction (PPI) analysis, comparing interregional couplings in late learning trials (mean S–R–O Repetitions 7 and 8) and in early learning trials (mean S–R–O Repetitions 2 and 3). Voxels colored in blue (Putamen and anterior Hippocampus) depict significant correlations across subjects between the PPI effect and the behavioral index of O–R encoding strength. Voxels colored in red (anterior Caudate and posterior Hippocampus) depict significant correlations between the PPI effect and the behavioral index of active O–R usage. For visualization purposes, the images were thresholded at *p* < .005 uncorrected.

**Fig. 5:**
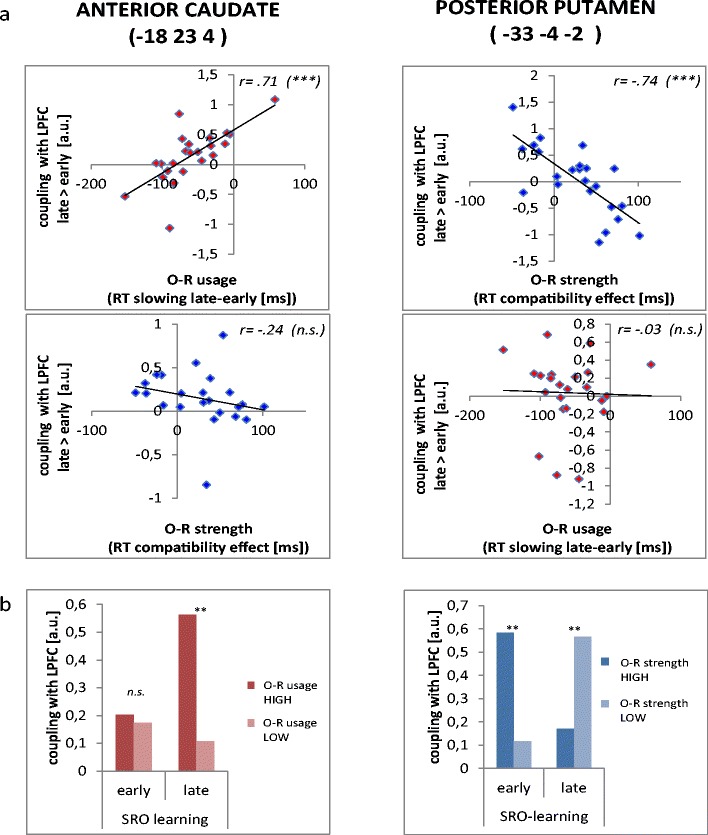
Summary of the results obtained by the left lateral prefrontal cortex (LPFC)-seeded (MNI –42 8 28) PPI analysis for two exemplary clusters exhibiting significant correlations with the behavioral indices of either active O–R usage (i.e., RT slowing; red/light grey in print) or O–R encoding strength (i.e., O–R compatibility effect; blue/dark grey in print). (A) Scatterplots depict correlations between the behavioral indices and the gPPI effects for late versus early learning trials. (B) The bar graphs depict the size of the gPPI effects in early and late learning trials, shown separately for two groups of subjects defined by median splits according to O–R usage (left) and O–R encoding strength (right).

The analysis of primary importance assessed potential correlations between changes in LPFC couplings and the two behavioral covariates. A learning-related increase in the functional coupling between LPFC and bilateral anterior caudate was positively correlated with response slowing as the putative maker of active O–R usage, but not with the O–R compatibility effect as a putative marker of O–R encoding (see Table 2 and Figs. 4 and 5). The positive correlation between response slowing and LPFC–caudate coupling evolved during learning, with a significant effect late in learning but no significant effect early in learning. This pattern was confirmed by a median split sorting subjects into two groups according to the size of the O–R usage index (Fig. 5B). Moreover, the same correlational pattern was observed for the left posterior hippocampus (note that a similar, but nonsignificant, effect was observed in the homologous right posterior hippocampus). Supplementary Table S1 additionally includes the correlation coefficients for the association between response slowing and functional couplings for mid learning trials in relation to early and late learning trials. For all identified areas, this complementary analysis demonstrates a gradual increase from early to late with mid-learning correlations always lying between these two values. This means that the observed effects were not just driven by differences in functional couplings between guided (SRO-Rep1/2/3) and unguided (SRO-Rep4/5/6/7/8) learning trials, which might have been potentially associated solely with differences in S–R retrieval demands.

**Table 2:** Correlation between O–R usage during S–R–O learning and functional coupling with LPFC during S–R–O learning (late > early)

Region of interest	Subregion	MNI coordinates			Coupling at late – early						Coupling at early		Coupling at late	
					Covariate O–R usage				Covariate O–R strength	Comparison between covariates	Covariate O–R usage	Covariate O–R strength	Covariate O–R usage	Covariate O–R strength
		*x*	*y*	*z*	*t*	*p* Cluster (FWE-Corr.)	Cluster Size	*p* Voxel (FWE-Corr.)	*t* (*p* Uncorr.)	*t* (*p* Uncorr.)	*t* (p Uncorr.)	*t* (*p* Uncorr.)	*t* (*p* Uncorr.)	*t* (*p* Uncorr.)
Left basal ganglia	Ant. caudate	–15	23	4	4.90	.01	27	.02	–1.17 (n.s.)	4.94^***^	–0.55 (n.s.)	–0.36 (n.s.)	3.63^***^	–1.59 (n.s.)
Right basal ganglia	Ant. caudate	12	23	4	5.06	.01	17	.04	0.21 (n.s.)	3.94^***^	–0.75 (n.s.)	–0.45 (n.s.)	3.05^**^	–0.39 (n.s.)
Left hippocampus	Post. hippocampus	–33	–34	–8	3.92	.04	3	.05	–1.02 (n.s.)	4.05^***^	–1.24 (n.s.)	–0.38 (n.s.)	3.27^***^	–1.51 (n.s.)
Right hippocampus	Post. hippocampus	33	–34	–8	3.09	.17	4	.19	–0.92 (n.s.)	3.29^***^	–1.02 (n.s.)	0.22 (n.s.)	2.92^**^	–0.95 (n.s.)

^*^ *p* < .05; ^**^ *p* < .01; ^***^ *p* < .001; (n.s.) not significant

In contrast to O–R usage, O–R encoding strength by itself (i.e., size of the postlearning O–R compatibility effect) was correlated with a different set of functional couplings, and this correlation was structured in a completely different way (see Table 3 and Figs. 4 and 5). Specifically, O–R encoding strength was negatively correlated with LPFC–putamen functional coupling late in learning, and additionally positively correlated with LPFC–putamen functional coupling early in learning. Again, this pattern was confirmed by a median split sorting subjects into two groups according to the size of the O–R encoding-strength index (Fig. 5B). The same correlational pattern was also observed for the left and right anterior hippocampi. Supplementary Table S2 additionally includes the correlation coefficients for the association between O–R compatibility and functional couplings for mid learning in relation to early and late in learning. For all identified areas, this complementary analysis demonstrates a gradual decrease from early to late, with mid-learning correlations always lying between these two values. Again, this means that the observed effects were not primarily driven by differences in functional couplings between guided (SRO-Rep1/2/3) and unguided (SRO-Rep4/5/6/7/8) learning trials.

**Table 3:** Correlation between O–R strength and functional coupling with LPFC during S–R–O learning (late > early)

Region of interest	Subregion	MNI coordinates			Coupling at late – early						Coupling at early		Coupling at late	
					Covariate O–R strength				Covariate O–R usage	Comparison of covariates	Covariate O–R strength	Covariate O–R usage	Covariate O–R strength	Covariate O–R usage
		*x*	*y*	*z*	*t*	*p* (FWE-Corr.)	Cluster size	*p* Cluster (FWE-Corr.)	*t* (*p* Uncorr.)	*t* (*p* Uncorr.)	*t* (*p* Uncorr.)	*t* (*p* Uncorr.)	*t* (*p* Uncorr.)	*t* (*p* Uncorr.)
Left basal ganglia	Putamen	–33	–4	–2	–5.36	.005	135	.001	–0.15 (n.s.)	4.42^***^	2.80^**^	0.65 (n.s.)	–3.08^**^	0.55 (n.s.)
	Putamen	–27	–10	4	–4.93	.012	Same cluster as above		–0.43 (n.s.)	3.83^***^	2.99^**^	0.17 (n.s.)	–3.17^***^	–0.37 (n.s.)
	Putamen	–18	5	–8	–4.5	.026	Same cluster as above		–0.42 (n.s.)	3.48^***^	3.67^***^	1.67 (n.s.)	–2.56^**^	0.87 (n.s.)
Right basal ganglia	Putamen	36	–1	–2	–4.19	.048	19	.031	0.38 (n.s.)	3.86^***^	2.72^**^	0.45 (n.s.)	–1.56 (n.s.)	0.88 (n.s.)
Left hippocampus	Ant. hippocampus	–27	–13	–20	–4.33	.020	8	.035	–0.20 (n.s.)	3.51^***^	2.52^**^	0.86 (n.s.)	–2.35^**^	0.70 (n.s.)
Right hippocampus	Ant. hippocampus	18	–13	–17	–6.02	.001	20	.017	–2.06^*^	3.43^***^	2.78^**^	1.07 (n.s.)	–3.60^***^	–1.13 (n.s.)
	Ant. hippocampus	30	–19	–8	–4.83	.008	11	.029	0.45 (n.s.)	4.46^***^	2.59^**^	1.05 (n.s.)	–3.49^***^	1.47 (n.s.)

^*^ *p* < .05; ^**^ *p* < .01; ^***^ *p* < .001; (n.s.) not significant

Finally, we found no significant correlation between the behavioral indices and learning-related BOLD activation in striatal or orbitofrontal areas without considering their functional coupling with LPFC. A direct correlation with O–R strength was observed only for the most dorsal section of the left and right hippocampi [MNI –18 –37 1 and MNI 21 –34 4, *t* = 4.78; *p*(FWE) < .015 and *t* = 7.1, *p*(FWE) < .001]. But these hippocampal activation clusters were entirely nonoverlapping with the posterior hippocampal regions that exhibited a correlation between LPFC coupling and O–R usage.

### Control analyses

Two additional analyses were performed in order to exclude the possibility that any of the above reported correlations between functional couplings and the behavioral indices of O–R usage and O–R encoding strength might equally well be explained by interindividual differences in error rates related to S–R learning (see also the third step of the second-level fMRI analysis in the Method section). In general, the opposite seems to be true. Simultaneously entering response slowing and error rate difference (late vs. early) as covariates slightly increased rather than decreased the *t* values for the response-slowing covariate (Supplementary Table S3). The error rate covariate did not reach significance after small-volume FWE correction. Simultaneously entering the two covariates O–R compatibility and error rate difference (late vs. early) substantially increased rather than decreased the *t* values for the O–R compatibility covariate (Supplementary Table S4). This suggests that interindividual differences in error rates and O–R compatibility effects bind different variance components in the relevant brain areas that would otherwise be part of the residual, and hence would reduce *t* values. This interpretation is further corroborated by the finding that the error rate difference (late vs. early) significantly correlated with the learning-related coupling between LPFC and areas within putamen and anterior hippocampus that are highly similar to those found for the O–R compatibility covariate (Table 4).

**Table 4:** Correlation between S–R error rate and functional coupling with LPFC during S–R–O learning (late > early), controlled for O–R encoding strength

Region of interest	Subregion	MNI coordinates			Covariate S–R error rate difference late – early (controlled for O–R strength)						Covariate S–R error rate early (controlled for O–R strength)		Covariate S–R error rate late (controlled for O–R strength)	
					Coupling at late – early				Coupling at early	Coupling at late	Coupling at early	Coupling at late	Coupling at early	Coupling at late
		*x*	*y*	*z*	*t*	*p* (FWE-Corr.)	Cluster size	*p* Cluster (FWE-Corr.)	*t* (*p* Uncorr.)	*t* (*p* Uncorr.)	*t* (*p* Uncorr.)	*t* (*p* Uncorr.)	*t* (*p* Uncorr.)	*t* (*p* Uncorr.)
Left basal ganglia	Putamen	–30	–4	–5	4.72	.019	36	.012	–2.24^*^	2.19^*^	2.07^*^	0.46 (n.s.)	–0.94 (n.s.)	2.53^**^
	Putamen	–33	8	–5	4.25	.044	Same cluster as above		–2.65^**^	1.48 (n.s.)	4.88^***^	1.47 (n.s.)	–0.39 (n.s.)	2.49^*^
	Putamen	–30	–16	1	3.93	.077	Same cluster as above		–2.09^*^	2.10^*^	3.18^**^	–0.15 (n.s.)	–0.41 (n.s.)	1.95^*^
Right basal ganglia	Putamen	24	11	–8	4.56	.026	15	.039	–1.57 (n.s.)	3.76^***^	–1.13 (n.s.)	0.10 (n.s.)	–0.87 (n.s.)	3.81^***^
	Putamen	33	–10	–5	4.21	.049	9	.058	–2.25^*^	3.33^**^	2.53^**^	–0.69 (n.s.)	–0.75 (n.s.)	2.61^**^
Left hippocampus	Ant. hippocampus	–30	–7	–20	5.57	.002	47	.004	–3.84^***^	2.07^*^	2.55^**^	0.21 (n.s.)	–1.74 (n.s.)	2.19^*^
	Ant. hippocampus	–30	–7	–14	5.50	.002	Same cluster as above		–3.11^**^	2.49^*^	2.09^*^	0.53 (n.s.)	–1.54 (n.s.)	2.92^**^
Right hippocampus	Ant. hippocampus	33	–7	–14	4.29	.023	20	.016	–2.73^**^	2.26^*^	2.81^**^	–0.50 (n.s.)	–0.74 (n.s.)	2.55^**^
	Ant. hippocampus	36	–13	–11	4.16	.029	Same cluster as above		–2.52^**^	2.41^*^	3.17^**^	–0.54 (n.s.)	–0.72 (n.s.)	1.95^*^

^*^ *p* < .05; ^**^ *p* < .01; ^***^ *p* < .001; (n.s.) not significant

## Discussion

This study was set up to characterize the relationship between behavioral and neural indices of model-based (here: instruction-based) learning of novel goal-directed actions. Specifically, by combining the two data modalities, we aimed to gain novel insights about possible subprocesses of integrating O–R contingencies into the current task model that are difficult to obtain by separately assessing behavioral and neural data in isolation. One specific focus was on the anterior caudate, a region implicated in goal-directed behavior in feedback-driven learning situations (O’Doherty et al., 2004; Tanaka, Balleine, & O’Doherty, 2008; Tricomi, Delgado, & Fiez, 2004) and tightly coupled to the LPFC in instruction-based learning situations, especially with high rather than low O–R contingency (Ruge & Wolfensteller, 2013).

In the present study, we found clear indication that active O–R usage (increasing response slowing across S–R–O learning) was specifically associated with increasing learning-related functional coupling between the LPFC and anterior caudate. By contrast, O–R encoding strength by itself (test-phase O–R compatibility effect in response times) was linked to the coupling between LPFC and putamen (i.e., the rodent dorsolateral striatum), which constitutes the putative striatal subdivision held to be responsible for habit formation (Balleine & Ostlund, 2007; Yin & Knowlton, 2006) at least during intermediate stages of automatization (Ashby et al., 2010). Similar to the functional differentiation of striatal couplings with the LPFC, we also found distinct hippocampal areas that were coupled with LPFC as a function of either O–R usage (posterior hippocampus) or O–R encoding strength (anterior hippocampus).

Importantly, none of the mentioned correlations between LPFC-seeded couplings and O–R encoding strength or O–R usage could be reduced to interindividual variability in S–R learning as indexed by error rates. On the contrary, variability in S–R learning and variability in O–R learning seem to be independently associated with couplings between LPFC and almost identical locations in putamen and hippocampus under instruction-based learning conditions. Thus, although S–R learning and O–R learning seem to share common neural substrates, the engagement of these shared neural resources for S–R learning and O–R learning is dissociated across subjects (e.g., a good early S–R learner is not necessarily a good early O–R learner and vice versa). This independence of correlations might be surprising on the one hand, as O–R learning can be regarded as being closely related to S–R learning (cf. Balleine & Ostlund, 2007). In both cases a mental representation of a stimulus becomes associated with a response. However, on the other hand, it is also clear that additional sources of variability might affect O–R learning even though the O is essentially also just a stimulus. First, the S in S–R learning is sensorially present prior to responding whereas the O is only mentally represented prior to responding. Second, to become mentally represented in the first place, the O needs be retrieved through S–O associations that must have been learned beforehand. Both aspects might add across-subjects variability specifically in O–R learning that is not present in S–R learning.

Another important observation is that, without considering functional couplings to the LPFC, neither caudate nor putamen were correlated with the behavioral indices of O–R strength or O–R usage, respectively. This is generally a crucial point to make as the striatal dependency on LPFC activation is exactly what is expected for theoretical reasons when learning is based on an explicit model of O–R contingencies temporarily being stored in the LPFC (Doll et al., 2009; Doll et al., 2012; Ramamoorthy & Verguts, 2012). However, it should be noted that the type of connectivity analysis chosen here does not warrant unequivocal statements about causal relationships. Hence, it might well be that the functional couplings between LPFC and striatal areas are in fact driven by common input from other areas. For instance, O–R learning might take place in the putamen as well as in the LPFC, but both might be only indirectly coupled via a common driving input from S–O encoding processes that might possibly be mediated by orbitofrontal regions, especially for nonincentive outcomes like the ones employed in the present study (Clark, Hollon, & Phillips, 2012; McNamee, Rangel, & O’Doherty, 2013).

Furthermore, the observed fronto-striatal couplings were not only differentially correlated with O–R usage and O–R encoding, but the temporal structure of these correlations was entirely different. O–R usage was positively correlated with LPFC–caudate coupling late in learning but not yet early in learning. This might simply suggest that it takes a few learning cycles before the full S–R–O contingency model is being integrated into caudate-based action selection processes. These “few learning cycles” do not seem to be directly related to our O–R encoding index, which was neither correlated with the behavioral O–R usage index nor modulated the correlation between O–R usage and LPFC–caudate coupling. Hence, although it is a necessary element in the putative S-O → O–R chain, successful O–R encoding alone is not sufficient for O–R usage.

The correlational dynamics regarding LPFC–putamen coupling were different. Specifically, O–R encoding strength was negatively correlated with LPFC–putamen coupling late in learning and additionally positively correlated with LPFC–putamen coupling early in learning. This seems to suggest that good O–R learners (indicated by strong O–R compatibility effects) benefited from an early boost in LPFC–putamen coupling leaving quite some time for subsequent O–R consolidation. By contrast, the late boost of the same couplings in weak O–R learners might imply less remaining time for O–R consolidation, which in sum results in relatively poorly encoded O–R associations. Interestingly, the LPFC showed a similar temporal pattern of couplings with quite similar areas in the putamen (and anterior hippocampus, for that matter) as a function of the error rate difference between late and early stages in learning. Specifically, when the error rate was relatively high late (i.e., small error rate difference relative to early learning trials), LPFC–putamen coupling was relatively weak early and relatively strong late. By contrast, when the error rate was relatively low late, LPFC–putamen coupling was relatively strong early and relatively weak late. Together this suggests that the LPFC–putamen coupling increases whenever S–R learning is ongoing. In some subjects, S–R learning occurs early (accompanied by strong coupling) resulting in strongly reduced error rates late (accompanied by weak coupling). Other subjects show weak S–R learning early (accompanied by weak coupling) and have to catch up with S–R learning late (accompanied by strong coupling).

### Specificity of LPFC–anterior caudate functional couplings

Together, the present findings clearly suggest that the functional coupling between LPFC and anterior caudate is specifically linked to active O–R usage rather than O–R encoding by itself. Our findings go beyond previous studies that demonstrated an involvement of anterior caudate in instruction-based learning but did not look into possible functional couplings (Ruge & Wolfensteller, 2010; Stocco, Lebiere, O’Reilly, & Anderson, 2012), or that demonstrated functional couplings between PFC and caudate but did not establish a clear link with the expression of overt goal-directed behavior (Li et al., 2011; Ruge & Wolfensteller, 2013). It is interesting to note that the observed correlation between O–R usage and LPFC-seeded functional coupling was located rather dorsally in the anterior caudate (*z* = +4). This corresponds roughly to a “dorsal striatum” region (*z* =0) that has previously been interpreted to be relevant for linking predicted outcomes to actions under trial-and-error learning conditions (O’Doherty et al., 2004). By contrast the same previous study implicated the “ventral striatum” region (*z* = –10) in stimulus-based outcome prediction. In the present study the ventral striatum (*z* = –8) exhibited a generally increased learning-related coupling with the LPFC without being modulated by O–R usage. Hence together, this might suggest that S–O prediction supported by the ventral anterior caudate region is relatively stable across subjects and only action selection based on that predicted outcome varies considerably between subjects.

Generally, the convergence of results between trial-and-error learning and instruction-based learning highlights the strikingly similar functional differentiation within the anterior striatum. Yet, the important distinction seems to be that in the latter case it emerges through functional coupling with the LPFC.

### Stimulus–response associations, outcome–response associations, and the habit concept

The putamen is typically thought to be part of the habit system (Ashby et al., 2010; Dolan & Dayan, 2013; Seger & Spiering, 2011; Yin & Knowlton, 2006). Accordingly, the association between LPFC–putamen coupling and S–R encoding (error rate index) and O–R encoding (O–R compatibility index) suggests an early formation of habit-like S–R and O–R memory traces under instructed learning conditions. These observations are in line with recent re-assessments of certain aspects of the traditional habit concept (e.g., Seger & Spiering, 2011). First, it is well known that behavioral control shifts from goal-directed to habitual with extended practice. Contrary to this notion, in the present study the extent of practice was minimal (eight S–R–O repetitions). It is therefore likely that putamen involvement in the present context reflected not the behavioral control by habit-like representations, which would become increasingly dominant only with extended practice, but rather the initial formation of habit-like representations. Hence, learning-related LPFC–putamen coupling seems to reflect the initial formation of S–R and O–R representations as precursors of “true” habits, that would develop if practice would proceed substantially longer up to a point at which the putamen might act independently of the lateral PFC. This is in line with the classical distinction between latent learning and performance (Tolman, 1948) and with results showing that deactivations of certain cortical regions in the rodent brain can shift behavioral control back-and-forth between habitual and goal-directed independent of the amount of practice (Killcross & Coutureau, 2003). Generally, increasing evidence seems to support a concurrent engagement of both habit-related and goal-related computations early in practice (Brovelli, Nazarian, Meunier, & Boussaoud, 2011; Dolan & Dayan, 2013; Seger & Spiering, 2011).

Second, a habit is typically thought to be operating automatically and in the absence of voluntary control based on explicit knowledge buffered in the PFC. Contrary to this notion, in the present study the neural instantiation of precursors of both “S–R habits” and “O–R habits” emerged specifically via LPFC–putamen interaction suggesting an involvement of explicit contingency knowledge stored in the LPFC. Based on this we suggest the possibility that habits might be established via two different mechanisms. The classical mechanism would operate in the absence of awareness, completely model-free and solely relying on the putamen. Alternatively, habit (S–R as well as O–R) formation in the putamen might occur in the presence of awareness, scaffolded by an explicit model held in the LPFC. From a broader perspective, this characterization of LPFC function in rule-guided behavior is, for instance, in line with data showing that the LPFC is becoming engaged once a subject becomes aware of “hidden” (i.e., relatively complex) event contingencies (Rose, Haider, & Büchel, 2010).

### Functional couplings between LPFC and hippocampus

Although previous studies have already demonstrated that instruction-based influences on learning are associated with fronto-hippocampal interactions (Li et al., 2011; Ruge & Wolfensteller, 2013), the present dissociation between hippocampal subregions appears more difficult to account for conceptually than the dissociation between caudate and putamen discussed above. However, the hippocampus has recently been suggested to play an important role in predicting future states and this hippocampal capacity has in fact been linked to the goal-directed control of action (Fouquet et al., 2013; Johnson, van der Meer, & Redish, 2007; Kennedy & Shapiro, 2009; Zilli & Hasselmo, 2008). Indeed it seems that—consistent with our results—a number of such studies have specifically implicated the posterior/dorsal hippocampus in goal-directed action control (Fouquet et al., 2013; Gourley, Lee, Howell, Pittenger, & Taylor, 2010).

More generally, the hippocampus is known to play a pivotal role in experience-based single-trial episodic memory formation and retrieval (e.g., O’Reilly & Rudy, 2001; Rolls, 2010; Tulving & Markowitsch, 1998). Importantly, there is also ample evidence for an interaction between working memory and episodic memory processes that seems to be based on an interaction between LPFC and hippocampal/parahippocampal areas (Bergmann, Rijpkema, Fernández, & Kessels, 2012; Meyer-Lindenberg et al., 2005; Ranganath, Cohen, & Brozinsky, 2005; Zilli & Hasselmo, 2008). For instance, anterior hippocampus activation at the time point of encoding has been shown to differentiate particularly well between subsequent correct and erroneous recall of an experienced episode (Summerfield et al., 2006). Notably, the same study also showed that the functional coupling between LPFC and anterior hippocampus predicted subsequent memory performance. Such a finding is in line with the present study results, which suggest a similar relevance of LPFC coupling with the anterior hippocampus during encoding for the subsequent retrieval of O–R associations as expressed in the size of the O–R compatibility effect. This coupling might indicate either the transfer of O–R working memory representation into episodic long-term memory (e.g., Ranganath et al., 2005) or it might reflect the integration of episodic O–R memories into the current task model stored in LPFC working memory.

Interestingly, two other studies reported activations in the anterior hippocampus without LPFC coupling when extensively learnt response outcomes served as primes that should automatically trigger response tendencies consistent with previously established O–R associations (Elsner et al., 2002; Melcher et al., 2013). This suggests that the anterior hippocampus might become independent of LPFC after extensive learning of O–R associations and is not exclusively relevant for short-term O–R encoding/retrieval processes accomplished in cooperation with LPFC as is the case under instruction-based learning conditions in the present study.

## Conclusions

Together, our results suggest that fronto-striatal couplings enable the learning of novel goal-directed actions via an internal model of response–outcome contingencies buffered in LPFC “procedural working memory” (cf. Oberauer, 2009). More specifically, the learning-related functional coupling between LPFC and anterior caudate is associated with O–R usage rather than with O–R encoding by itself. This suggests a pivotal role of the anterior caudate for establishing the online control of goal-directed action and implies a similar striatal specialization as in model-free feedback-driven learning, yet emerging through functional couplings with the LPFC. As another novel contribution, our study shows that hippocampal involvement in goal-directed action also emerges through functional couplings with the LPFC, and that this hippocampal involvement can be differentiated according to O–R usage (posterior/dorsal) and O–R encoding (anterior/ventral). It remains to be clarified whether the reported correlational effects between behavior and functional couplings are specific for instruction-based learning situations or would generalize to situations in which novel rules are entirely and solely extracted via trial and error.

## Electronic supplementary material

Below is the link to the electronic supplementary material.

Supplementary Table S1 (PDF 39 kb)

Supplementary Table S2 (PDF 42 kb)

Supplementary Table S3 (PDF 42 kb)

Supplementary Table S4 (PDF 44 kb)

## Footnotes

1 Note that differential outcomes affect learning and performance in both trial-and-error S–R learning and instruction-based S–R learning, even though they are strictly speaking not necessary for correct performance. In fact, differential outcomes are not even a necessary condition for goal-directed action at all. Just implementing a novel S–R rule is sufficient to qualify as a goal-directed action, due to the “hidden” outcome anticipation of receiving correct feedback as the rewarding event for a correctly performed action (see also Ruge & Wolfensteller, 2013; Wolfensteller & Ruge, 2012). However, differential outcomes, be they incentive or not (Friedrich & Zentall, 2011; Mok & Overmier, 2007), serve as additional “pointers” or retrieval cues for this basic relationship between S, R, and the incentive outcome (here: “correct feedback”), and thus are an integral part of the associational basis of goal-directed action. For the present purposes, the differential-outcome rationale is a means to access action–outcome integration in behavior and brain activation.

2 A previous behavioral study comprised an additional control condition in which all responses were followed by one common outcome sound, rather than differential outcome sounds, as in the present study (Ruge et al., 2012). Thereby, we could demonstrate more directly that the presence of differential outcomes during rapid S–R learning is associated with response slowing (but not error rates) on top of the general response speed-up related to S–R learning.
