# Supplementary material for: Distinct fronto-striatal couplings reveal the double-faced nature of response–outcome relations in instruction-based learning
Source: Cogn Affect Behav Neurosci. 2014 Nov 1;15(2):349–64. doi: 10.3758/s13415-014-0325-4 (PMC4436102; doi:10.3758/s13415-014-0325-4)
Supplement: Supplementary file 1 — (PDF 39 kb) [file 13415_2014_325_MOESM1_ESM.pdf]

Table S1. Correlation between O-R usage during S-R-O learning and functional coupling with LPFC during S-R-O learning (early, middle, late)

Region of Interest	Sub-region	MNI coordinates			covariate O-R usage					
					coupling at late - early	coupling at middle - early	coupling at late - middle	coupling at early (SR23)	coupling at middle (SR56)	coupling at late (SR78)
		x	y	z	r (p uncorr.)	r (p uncorr.)	r (p uncorr.)	r (p uncorr.)	r (p uncorr.)	r (p uncorr.)
Left basal ganglia	ant. caudate	-15	23	4	0.71***	0.14 (n.s.)	0.52**	-0.12 (n.s.)	0.04 (n.s.)	0.61**
Right basal ganglia	ant. caudate	12	23	4	0.72***	0.34 (n.s.)	0.25 (n.s.)	-0.16 (n.s.)	0.26 (n.s.)	0.54**
Left hippocampus	post. hippocampus	-33	-34	-8	0.63***	0.40*	0.40*	-0.26 (n.s.)	0.12 (n.s.)	0.57**
Right hippocampus	post. hippocampus	33	-34	-8	0.55**	0.32 (n.s.)	0.27 (n.s.)	-0.21 (n.s.)	0.25 (n.s.)	0.53**

* p<.05; ** p<.01; *** p<.001; (n.s.) not significant
