# Supplementary material for: Distinct fronto-striatal couplings reveal the double-faced nature of response–outcome relations in instruction-based learning
Source: Cogn Affect Behav Neurosci. 2014 Nov 1;15(2):349–64. doi: 10.3758/s13415-014-0325-4 (PMC4436102; doi:10.3758/s13415-014-0325-4)
Supplement: Supplementary file 2 — (PDF 42 kb) [file 13415_2014_325_MOESM2_ESM.pdf]

Table S2. Correlation between O-R strength and functional coupling with LPFC during S-R-O learning (early, middle, late)

Region of Interest	Sub-region	MNI coordinates			covariate O-R strength					
		x	y	z	coupling at late - early r (p uncorr.)	coupling at middle - early r (p uncorr.)	coupling at late - middle r (p uncorr.)	coupling at early (SR23) r (p uncorr.)	coupling at middle (SR56) r (p uncorr.)	coupling at late (SR78) r (p uncorr.)
Left basal ganglia	Putamen	-33	-4	-2	-0.74***	-0.50**	-0.43*	0.51**	-0.10 (n.s.)	-0.55**
	Putamen	-27	-10	4	-0.72 ***	-0.44**	-0.49**	0.53**	-0.10 (n.s.)	-0.56**
	Putamen	-18	5	-8	-0.68***	-0.41*	-0.54**	0.61***	0.12 (n.s.)	-0.48*
Right basal ganglia	Putamen	36	-1	-2	-0.66***	-0.49**	-0.06 (n.s.)	0.50**	-0.23 (n.s.)	-0.32 (n.s.)
Left hippocampus	ant. hippocampus	-27	-13	-20	-0.67***	-0.42*	-0.48*	0.42*	0.04 (n.s.)	-0.45*
Right hippocampus	ant. hippocampus	18	-13	-17	-0.77***	-0.42*	-0.45*	0.51**	-0.04 (n.s.)	-0.60**
	ant. hippocampus	30	-19	-8	-0.71***	-0.65***	-0.40*	0.48**	-0.42*	-0.59**

* p<.05; ** p<.01; *** p<.001; (n.s.) not significant
