# Supplementary material for: Distinct fronto-striatal couplings reveal the double-faced nature of response–outcome relations in instruction-based learning
Source: Cogn Affect Behav Neurosci. 2014 Nov 1;15(2):349–64. doi: 10.3758/s13415-014-0325-4 (PMC4436102; doi:10.3758/s13415-014-0325-4)
Supplement: Supplementary file 3 — (PDF 42 kb) [file 13415_2014_325_MOESM3_ESM.pdf]

Table S3. Correlation between O-R usage during S-R-O learning and functional coupling with LPFC during S-R-O learning (late>early) controlled for S-R error rate difference late-early

Region of Interest	Sub-region	MNI coordinates			covariate O-R usage (controlled for S-R error rate difference late-early)					
					coupling at late - early				coupling at early	coupling at late
		x	y	z	t	p voxel (FWE-corr.)	cluster size	p cluster (FWE-corr.)	t (p uncorr.)	t (p uncorr.)
Left basal ganglia	ant. caudate	-12	23	4	6.75	0.001	42	0.01	-0.94 (n.s.)	3.84***
		-12	20	-5	4.18	0.045	same cluster as above		-0.56 (n.s.)	2.49*
Right basal ganglia	ant. caudate	12	26	4	5.47	0.004	26	0.02	-0.51 (n.s.)	3.01**
		6	20	4	5.17	0.008	same cluster as above		-1.70 (n.s.)	1.59 (n.s.)
Left hippocampus	post. hippocampus	-33	-34	-8	4.34	0.019	10	0.03	-1.24 (n.s.)	3.63***
Right hippocampus	post. hippocampus	36	-34	-8	3.79	0.053 (n.s.)	2	0.058 (n.s.)	-1.15 (n.s.)	3.25**

* p<.05; ** p<.01; *** p<.001; (n.s.) not significant
