# Supplementary material for: Distinct fronto-striatal couplings reveal the double-faced nature of response–outcome relations in instruction-based learning
Source: Cogn Affect Behav Neurosci. 2014 Nov 1;15(2):349–64. doi: 10.3758/s13415-014-0325-4 (PMC4436102; doi:10.3758/s13415-014-0325-4)
Supplement: Supplementary file 4 — (PDF 44 kb) [file 13415_2014_325_MOESM4_ESM.pdf]

Table S4. Correlation between O-R encoding strength and functional coupling with LPFC during S-R-O learning (late>early) controlled for S-R error rate difference late-early

Region of Interest	Sub-region	MNI coordinates			covariate O-R strength (controlled for S-R error rate difference late-early)					
					coupling at late - early				coupling at early	coupling at late
		x	y	z	t	p (FWE-corr.)	cluster size	p cluster (FWE-corr.)	t (p uncorr.)	t (p uncorr.)
Left basal ganglia	Putamen	-30	-4	-5	-8.30	0.001	330	0.001	4.12***	-3.68***
	Putamen	-30	-16	1	-6.46	0.001	same cluster as above		3.04**	-3.83***
	Putamen	-18	5	-8	-6.19	0.001	same cluster as above		3.62***	-3.61***
Right basal ganglia	Putamen	30	-10	-5	-5.83	0.005	same cluster as above		3.30**	-4.00***
	Putamen	30	14	1	-5.04	0.011	245	0.001	2.74**	-3.59***
	Putamen	24	-1	-5	-4.71	0.020	same cluster as above		3.08**	-2.50**
Left hippocampus	ant. hippocampus	-30	-7	-14	-6.27	0.001	78	0.001	3.38**	-3.01**
	ant. hippocampus	-30	-13	-20	-6.03	0.001	same cluster as above		3.04**	-3.18**
Right hippocampus	ant. hippocampus	30	-19	-8	-6.67	0.001	125	0.001	2.27*	-5.50***
	ant. hippocampus	30	-4	-20	-4.88	0.008	same cluster as above		3.12**	-3.22**

* p<.05; ** p<.01; *** p<.001; (n.s.) not significant
